# The Early and Intensive Motor Training for Spinal Cord Injury Trial: key principles to consider when undertaking large international investigator-driven clinical trials

**DOI:** 10.1038/s41393-026-01232-y

**Published:** 2026-06-30

**Authors:** Lisa A. Harvey, Joanne V. Glinsky, Jackie Chu

**Affiliations:** 1 Kolling Institute, Faculty of Medicine and Health, The University of Sydney, Sydney, Australia; 2 John Walsh Centre for Rehabilitation Research, Northern Sydney Local Health District, Sydney, NSW Australia; 3 Repat Health Precinct, Daw Park, SA Australia; 4 Royal North Shore Hospital, St Leonards, NSW Australia; 5 Prince of Wales Hospital, Randwick, NSW Australia; 6 The Princess Alexandra Hospital, Brisbane, QLD Australia; 7 Sunnaas Rehabilitation Hospital, Nesodden, Norway; 8 Adelante Centre of Expertise in Rehabilitation and Audiology, Hoensbroek, Netherlands; and Department of Rehabilitation Medicine, Research School CAPHRI, Maastricht University, Maastricht, Netherlands; 9 Department of Rehabilitation Medicine, Leiden University Medical Centre, Leiden, Netherlands; 10 Center of Excellence for Rehabilitation Medicine, University Medical Center Utrecht Brain Center, University Medical Center Utrecht and De Hoogstraat Rehabilitation, Utrecht, the Netherlands; 11 Queen Elizabeth National Spinal Injuries Unit, Queen Elizabeth University Hospital, Glasgow, Scotland UK; 12 Royal Talbot Rehabilitation Centre, Austin Health, Kew, VIC Australia; 13 Ghent University Hospital, Ghent, Belgium; 14 London Spinal Cord Injury Centre, Royal National Orthopaedic Hospital Trust, London, Middlesex UK; 15 Royal Rehab, Putney, NSW Australia; 16 Fiona Stanley Hospital, Murdoch, WA Australia; 17 University Hospitals Leuven, Department of Physical and Rehabilitation Medicine, Leuven, Belgium; 18 “A. Alesini” Hospital, Rome, Italy; 19 REVAL, Hasselt University, Hasselt, Belgium; 20 I.R.C.C.S. Santa Lucia Foundation, Rome, Italy; 21 Department of Life Sciences, Health and Health Professions, Link Campus University, Rome, Italy

**Keywords:** Neurology, Medical research

## Abstract

**Study design:**

Methodological commentary.

**Objectives:**

To summarise key methodological and operational principles for designing and conducting large, international, investigator-driven clinical trials involving physical interventions for people with recent spinal cord injuries (SCI). This paper draws on our experience designing and conducting The Early and Intensive Motor Training for SCI Trial (SCI-MT Trial).

**Setting:**

15 spinal injury units across Australia, Scotland, England, Italy, the Netherlands, Norway, and Belgium.

**Methods:**

We reviewed the SCI-MT Trial protocol, documentation, and procedures to identify key methodological and operational issues relevant to large, international trials involving physical interventions for people with recent SCI. Guidance was developed based on both our practical experience and current recommendations from clinical trial experts, with the aim of informing and supporting future trialists.

**Results:**

We identified key principles that should be considered when designing and conducting trials of physical interventions for people with recent SCI. These included principles related to trial design (Methodological Principles) and trial conduct (Operational Principles).

**Conclusions:**

This paper provides guidance on the principles and practicalities of designing and implementing large, international, investigator-driven clinical trials. It is intended to assist future trialists in planning trials that are methodologically rigorous, operationally feasible, and ethically robust.

**Trial registration:**

ACTRN12621000091808 (1.2.2021).

**Trial identifier no:**

U1111-1264-1689.

**Protocol no:**

Version 1.3, 22nd Oct 2021.

## Introduction

We recently completed a trial titled: The Early and Intensive Motor Training for Spinal Cord Injury Trial (The SCI-MT Trial). The trial randomised 220 participants within 10 weeks of injury to either usual care or usual care plus an additional 12 h per week of intensive motor training administered for 10 weeks. This investigator-driven trial was conducted across 15 spinal injury units in 7 countries. This trial is among the largest trials of a complex physical rehabilitation intervention undertaken in people with recent spinal cord injury (SCI). The results showed no therapeutic effect of the additional Motor Training on the primary outcome (total motor score) at 10 weeks or any of the secondary outcomes related to mobility, function, or psychological status at either 10 weeks or 6 months. The only exception was a small improvement in the psychological domain of the WHOQOL-BREF at 10 weeks. The full results of this trial [1], along with the trial protocol and findings from related sub-studies, have been reported previously [1–8].

This paper summarises key methodological and operational principles that researchers should consider when designing and conducting similar trials. The guidance is intended as a practical resource, to provide guidance based on our experience, particularly in trials of physical interventions conducted in SCI units. Examples from the SCI-MT Trial are provided throughout to illustrate these principles in practice. Importantly, the SCI-MT Trial depended on the collaboration of participating SCI units. Unlike commercially funded trials, sites did not receive full financial support to cover all trial-related activities. For example, therapists not employed by the trial who delivered usual care, were asked to maintain simple records of all physiotherapy and occupational therapy sessions. This reliance shaped several aspects of trial design and management. Accordingly, the guidance provided here should be interpreted within this context. It should also be considered in light of the trial’s funding level (approximately $AUD 3.5 million). However, most of the key principles described are broadly applicable, even to smaller or less well-funded trials.

## Methods

We reviewed the SCI-MT Trial protocol, operational documentation, and trial management procedures to identify key methodological and operational principles that should be considered when designing and conducting similar trials (see Table 1 for summary). This review reflected discussions among investigators, trial coordinators, and site representatives during the conduct of the trial. For each principle, we developed practical guidance informed by the best available recommendations from clinical trial experts, existing literature, and regulatory or good-clinical-practice guidelines. These recommendations were further refined using our experience in designing, managing, and implementing the SCI-MT Trial across multiple international sites. We also drew on a related paper authored by our group in 2018 [9]. The guidance provided here aims to inform future trialists by outlining anticipated challenges and the procedural considerations required for large, multicentre trials involving physical interventions for people with SCI.

**Table 1:** A summary of the principles.

***METHODOLOGICAL PRINCIPLES***
**A. Overall design of the trial**
Formulate a research question with meaningful real-world impact Clarify whether the trial is pragmatic or explanatory Select inclusion criteria that identify participants most likely to benefit Intervention duration and intensity should be maximised where feasible Conduct a power analysis, recognising its limitations Incorporate process and economic evaluations from the design phase Ensure early planning for the CONSORT Flow Chart Register the trial prospectively and publish a detailed protocol
**B. The outcomes**
Select outcomes that reflect function, independence, or quality of life, and specify a single primary outcome Limit secondary outcomes and interpret them with caution Outcomes with limited reliability reduce precision but do not introduce systematic bias Prioritise collecting the primary outcome on as many participants as possible Capture participants’ impressions of therapeutic benefit and include open-ended questions about the intervention and its burden Ensure assessors are thoroughly trained in outcome data collection procedures Conduct baseline assessments prior to randomisation Schedule outcome assessments relative to randomisation, not the start of the intervention Blind assessors and verify the success of blinding Conceal allocation for those enrolling participants
**C. The intervention**
Describe the intervention in accordance with the TIDIER Guidelines Provide comprehensive training to intervention staff and ensure understanding of key principles Monitor intervention delivery to confirm fidelity
**D. Data management and trial integrity**
Confirm randomisation details to prevent allocation errors Design the Case Report Forms (CRFs) and database to minimise errors and simplify data entry Continuously verify data accuracy throughout the trial, not just at the end Develop clear procedures for recording Serious Adverse Events (SAEs) and Adverse Events (AEs) Maintain strict version control of study documents Use a Data Safety Monitoring Committee
**E. The analysis, interpretation and reporting of results**
Do not perform statistical tests on baseline data It is occasionally acceptable to exclude randomised participants from analysis Pre-specify all subgroup analyses Interpret mean between-group differences relative to a prespecified minimal important difference Changes over time within groups do not provide inherently useful information Interim trial results should not be reported except under predefined conditions Report all between-group results in a single publication and avoid spin Share trial results with all participants and stakeholders upon trial completion

***OPERATIONAL PRINCIPLES***
**F. Operational issues**
Assemble a skilled, collaborative team you trust Select sites with similar operations, values and funding models Be mindful of the additional workload placed on the SCI units Engage a reputable Clinical Research Organisation (CRO) but read the fine print and expect to pay for the service Allow substantial time for contracts and approvals especially with international sites Understand the country-specific data, consent, and confidentiality regulations Be prepared for additional challenges when conducting trials in many languages

## Methodological principles

### Overall design of the trial

#### Formulate a research question with meaningful real-world impact

The underlying research question is the most important aspect of any clinical trial [10], yet formulating a meaningful research question can be challenging. The question should have clinical relevance for a broad group of people, rather than focusing on minor details of intervention delivery that may be of narrow interest. For example, in the SCI-MT Trial our central question - whether intensive motor training improves neurological recovery - had clear real-world relevance because people with SCI are actively seeking high-dose training programs, and SCI units worldwide are striving to provide increasingly intensive therapy. The results therefore have direct implications for the type and extent of therapy delivered in routine practice. Closely linked to this consideration is the need to select a question that is feasible given the available resources. The research question should be clearly defined using the PICO framework - specifying the Population, Intervention, Comparison, and Outcome [11]. It is also essential to obtain support for the research question from both people with SCI and the clinicians or staff responsible for implementing the trial [12].

#### Clarify whether the trial is pragmatic or explanatory

Understanding whether a trial is pragmatic or explanatory has important implications for many aspects of trial design [13, 14]. Explanatory trials are designed to determine whether an intervention is effective under ideal conditions, whereas pragmatic trials aim to assess effectiveness when the intervention is implemented in real-world practice. The SCI-MT Trial was a pragmatic trial, which required the intervention to be delivered as it would be in routine clinical practice. For example, as happens in the real world, clinicians exercised their clinical judgment to tailor the intervention to each participant while being guided by key principles.

The distinction between pragmatic and explanatory trials extends beyond the intervention itself; it also affects decisions about the eligibility criteria, primary outcomes, type of analysis (intention-to-treat or per protocol) [15] and follow-up procedures. Often, the distinction is not absolute, and trials exist on a continuum between explanatory and pragmatic. Tools such as the Pragmatic–Explanatory Continuum Indicator Summary (PRECIS) can help researchers position their trials along this spectrum [16].

#### Select inclusion criteria that identify participants most likely to benefit

The inclusion criteria should reflect the participants most likely to respond to the intervention, although determining this is complex. Available evidence should guide these decisions. For example, in the SCI-MT Trial, participants were excluded if they had an AIS A lesion (unless they retained some motor function more than three levels below one of their two motor levels) and AIS B lesion because evidence suggests that spinal neuroplasticity is most apparent in individuals with some preserved motor function below the motor or neurological level. Additionally, only participants within 10 weeks of injury were included, as the spinal cord is believed to be most responsive to therapeutic intervention during this early period. Notably, even carefully considered inclusion criteria are not foolproof. If a trial yields a negative result, the criteria may be questioned, regardless of the rationale used to define them.

#### Intervention duration and intensity should be maximised where feasible

When a trial is not designed to evaluate dosage, providing the highest feasible dose may help minimise false-negative findings, recognising that the appropriate dose will vary across individuals, outcomes, and intervention modalities. It is important to avoid conducting a lengthy trial that ultimately yields a negative result due to insufficient intervention duration or intensity. If the trial produces a positive outcome, decisions about whether the intervention could be delivered at a lower dosage or for a shorter duration can be addressed in future non-inferiority trials [17].

#### Conduct a power analysis, recognising its limitations

A detailed power analysis is an essential component of trial planning, particularly for grant applications, where reviewers typically expect it. However, even with careful planning, power analyses often fall short and should not be regarded as exact predictions of the required sample size. They often underestimate the number of participants required for a trial. Power analyses rely on assumptions about the likely standard deviation (SD) which is difficult to predict. In the SCI-MT Trial, robust preliminary data were available to inform the initial power analysis. We estimated that the SD of the change in Total Motor Scores would be 13.3 points. In fact the SD was 10.3 points. Obtaining a smaller SD than anticipated is uncommon. Consequently, our estimate of the treatment effect (the 95% CI) was very precise and a smaller sample size would have been adequate. However, the SD cannot be predicted with precision in trials of this type, and it is generally preferable to overestimate rather than underestimate the required sample size even though larger samples increase costs and operational complexity. Once a trial has been completed, statistical power is no longer meaningful [18]. At this stage, the focus should be on the precision of the estimated treatment effect.

#### Incorporate process and economic evaluations from the design phase

Large trials often benefit from conducting process and economic evaluations in parallel with the main trial. Both require separate protocols, which should be made publicly available prior to trial commencement. A process evaluation uses multiple data sources to help explain trial results [3]. For example, in the SCI-MT Trial, we interviewed participants and therapists to examine their perceptions of the additional Motor Training and incorporated open-ended questions about the intervention in a trial-exit survey. These data were coded before the trial results were known. After determining that the trial results were negative, we examined the data for possible explanations. One potential explanation could have been that participants and therapists did not value the intervention and therefore were not fully engaged. However, the interviews (and other data sources) demonstrated that this was not the case and that both participants and therapists were highly engaged. We also collected detailed adherence and fidelity data to assess whether participants received the intended dosage and whether the intervention was delivered as planned [7].

Economic evaluations are particularly important for positive trial results, as they evaluate the cost implications of implementing the intervention more broadly. Critical decisions about the analyses must be made in advance, including whether they will be conducted from a consumer, health system, or societal perspective. Economic data collection can be demanding for participants, and excessive burden may result in incomplete or poor-quality data. It is therefore important to carefully weigh the advantages and disadvantages of different levels of data collection. Extensive economic data may provide limited insight when trial results are negative, as occurred in the SCI-MT Trial. Nonetheless, careful planning is required for both potential positive and negative trial outcomes. It is also imperative to include a health economist on the trial team to guide these decisions and analyses.

#### Ensure early planning for the CONSORT Flow Chart

A CONSORT Flow Chart is essential for the publication of trial results, including accurate data on the number of patients screened and subsequently randomised [19]. The screening population should be defined at the outset, as this determines who is included in the Flow Chart. For example, in the SCI-MT Trial, we screened only patients admitted to a site within three months of injury. Maintaining accurate screening logs can be resource-intensive for sites, but their accuracy is crucial. The most reliable approach is to collect screening logs routinely and monitor the number of participants screened at each site, ensuring these numbers align with expected admissions. Site monitors also have a responsibility to verify screening logs during site visits.

The CONSORT Flow Chart must also document the number of participants lost to follow-up, why they were lost and, importantly, the number of participants who contributed data for each outcome at each time point. Participants available at follow-up may still fail to provide data for specific outcomes. For example, participants may decline a questionnaire or data may be missing because of technical issues with testing equipment. It is therefore essential to clearly report how many participants contributed data for each outcome at every time point to ensure transparency and data integrity.

#### Register the trial prospectively and publish a detailed protocol

Prospective trial registration is now a requirement for most journals including Spinal Cord [20], meaning that the registration must be date-stamped before the first participant is randomised. To ensure accuracy and transparency, trial registrations should clearly specify how each outcome will be analysed, particularly when an outcome includes multiple components. For example, if the WHOQOL-BREF is included as an outcome, the registration should indicate the analyses will be conducted on its subdomains. The full trial protocol can be uploaded to the trial registry or hosted on a public server. For large or high-impact trials, it may also be appropriate to publish the protocol in a peer-reviewed journal. Trial protocols vary in length and level of detail, with comprehensive protocols often exceeding 100 pages and including guidance on all major operational decisions required throughout the trial. They should adhere to the SPIRIT guidelines [21]. For trialists who are new to protocol development, reviewing several detailed, published trial protocols can provide valuable insight into the level of detail required. If a protocol is amended, the change should be recorded in the trial registry at the time it occurs to maintain an accurate public record. In our trial, any protocol amendments were documented through the trial registry, where each change was date-stamped and accompanied by a short rationale to ensure transparency.

### The outcomes

#### Select outcomes that reflect function, independence, or quality of life, and specify a single primary outcome

In a pragmatic trial, more so than in an explanatory trial, the outcomes should capture what matters most to people with spinal cord injury (SCI). For trials evaluating physical interventions, this generally means the inclusion of measures that capture function, independence, or quality of life. Individuals with SCI are unlikely to engage in 12 h per week in an intervention that requires their hard work and dedication unless it has a tangible impact on these aspects of their lives. Neurophysiological changes are unlikely to be meaningful to people with SCI.

In the SCI-MT Trial, the primary outcome was total motor score. Although this measure is not intrinsically meaningful to people with SCI, it served as a proxy for functional outcomes such as walking ability and independence. We adopted this approach because impairment measures such as total motor scores typically exhibit less variability, reducing the required sample size. However, this strategy is only justified when there is a clear and indisputable relationship between the the impairment measure and meaningful aspects of function, independence, or quality of life, as is the case for strength and walking function.

Trialists should clearly define a single primary outcome upon which conclusions about the trial’s success will be based [22]. It is possible to use a composite primary outcome [23] although this comes with its own set of challenges and is not a widespread practice in SCI trials. Some authors use two primary outcomes (i.e., a co-primary outcome) but this also requires careful planning in case the results contradict each other. It may be appropriate to use co-primary outcomes for Type II Hybrid Implementation studies when you may have one clinical outcome and one implementation outcome: both reflecting very different aspects of the trial [24]. Co-primary outcomes need to be fully pre-specified, justified, and aligned with the trial’s objectives.

#### Limit secondary outcomes and interpret them with caution

There is a tendency to include numerous secondary outcomes, but this approach is not ideal for two key reasons. First, it places a substantial burden on participants, increasing the risk of dropouts, and potentially discouraging participation in future trials. The time required for participants to complete the full assessment should be evaluated, with careful consideration of participant burden. Second, including too many secondary outcomes increases the likelihood of spurious findings due to multiplicity. As a pragmatic guide, secondary outcomes can be limited to a modest set (for example, 8–12), noting that the field has not defined a specific numerical threshold. Importantly, each outcome intended for a between-group comparison counts individually; for example, the WHOQOL-BREF has four domains, each analysed separately, so they count as four outcomes, not one.

Secondary outcomes should encompass a range of measures that capture diverse aspects of relevance to people with SCI. However, secondary outcomes should always be interpreted cautiously, as they exist to help explain or contextualise the results of the primary outcome [25]. A single significant finding among numerous secondary analyses should not be taken as evidence of a treatment effect; rather, it should be discussed as potentially worthy of future investigation. For example, in the SCI-MT Trial, a significant improvement was observed at 10 weeks on the psychological domain of the WHOQOL-BREF. While this may suggest a psychological benefit of additional Motor Training, it would be inappropriate to overemphasise this isolated finding as justification for widespread implementation of the intervention.

#### Outcomes with limited reliability reduce precision but do not introduce systematic bias

Outcomes with established reliability are preferable; however, in the context of trials, reliability is less critical than is often assumed. High reliability improves the precision of effect estimates, whereas poor reliability reduces it and may increase the number of participants needed. Importantly, outcomes with lower reliability do not introduce systematic bias when measurement error is random (non-systematic) and the analysis uses a linear model applied to a continuous outcome [26]. Thus, a subjective outcome that is not highly reliable but captures aspects of importance to people with SCI can still be appropriate. In the SCI-MT Trial, participants rated, on a 10-point scale, their ability to perform goals identified at baseline. This outcome had strong construct validity but unknown reliability. While self-report may have introduced some bias, this was due to the reporting method, not the outcome’s unproven reliability. The key principle remains: limited reliability reduces the precision of effect estimates but does not introduce systematic bias.

#### Prioritise collecting the primary outcome on as many participants as possible

The primary outcome is the most important measure in a trial, and all efforts should be focused on obtaining it for as many participants as possible. Regardless of adherence, complications, or events occurring after randomisation, it is imperative to capture the primary outcome wherever feasible. The only reasons for not collecting a primary outcome from a participant are that the participant declines to provide it, becomes too ill, formally withdraws, or when continued contact is no longer appropriate or in the participant’s best interests, as determined by ethical oversight. Few other circumstances justify failure to collect the primary outcome.

#### Capture participants’ impressions of therapeutic benefit and include open-ended questions about the intervention and its burden

When standardised and objective outcome measures fail to detect a treatment effect, participants may still perceive that the intervention was meaningful or beneficial, as was the case in the SCI-MT Trial. This perception may reflect bias - for example, a desire to justify the substantial time and effort invested - or it may indicate that participants are experiencing effects not captured by conventional outcome measures. To address this, it is valuable to include a measure of global impression of therapeutic effect, asking participants to rate how much they feel they have changed since the start of the trial [27, 28]. Importantly, the same question must be asked of both control and intervention participants to account for changes due to usual care or natural recovery. Additionally, open-ended questions at trial exit can capture participant satisfaction and subjective improvements or benefits, though these responses are descriptive and should not be treated as formal outcomes. It may also be informative to ask participants to rate the burden of the intervention. For example, in the SCI-MT Trial, participants were asked to rate, on a 10-point scale, how burdensome they found the 12 h per week of training. The results indicated that participants were highly tolerant of the intervention. Although this finding had limited utility given the negative trial results, it provides useful insight for future studies and for understanding how demanding therapy is perceived by people with recent SCI.

#### Ensure assessors are thoroughly trained in outcome data collection procedures

All assessors must be appropriately trained. It is important to verify this by keeping detailed logs of all training provided to staff. This is used to ensure that only trained personnel conduct assessments. Compliance with this requirement should be verified during site monitoring visits. Training must be comprehensive, as outcome data collection can often involve procedural details that assessors must apply consistently. For example, in the SCI-MT Trial, we used two quality of life measures: the EQ-5D-5L and the WHOQOL-BREF. Both are self-report questionnaires, but the EQ-5D-5L asks participants to report on their health today, while some WHOQOL-BREF questions refer to experiences over the past two weeks. Assessors need to draw participants’ attention to these distinctions, as they can be easily missed. Additionally, it must be clear whether assessors need to directly observe participants performing activities for measures such as the SCIM or WISCI, or whether they can rely on participants’ or staffs’ reports on what participants can do. The critical point is that assessments are conducted consistently at each time point and are always performed by blinded assessors.

Unforeseen issues will arise, requiring procedural decisions. Any decision regarding an outcome measure should be made by a lead investigator blinded to group allocation and then communicated to all sites and assessors for future reference. In the SCI-MT Trial, to minimise potential errors, a page of instructions for each assessment was provided to assessors at every data collection session. We also maintained a Questions and Answers File that outlined all issues that arose. All sites had ongoing access to this file.

#### Conduct baseline assessments prior to randomisation

Baseline assessments are often used to increase the precision of effect estimates. For example, in the SCI-MT Trial, we collected baseline data for the primary and most secondary outcomes. Baseline assessments should ideally be conducted prior to randomisation for two main reasons. First, they preserve assessor blinding, as the participant has not yet been allocated to a group. More importantly, conducting baseline assessments prior to randomisation ensures that randomisation occurs as late as possible, minimising the risk of dropouts. Participants may become ill, withdraw consent or demonstrate intolerance to the assessments; any of these may preclude inclusion. Conducting baseline assessments before randomisation allows these issues to be identified and participants excluded if necessary, without compromising the integrity of the trial.

It is also essential to define, in the protocol, the acceptable time interval between baseline assessment and randomisation. For example, it is not appropriate to conduct a baseline assessment and then randomise a participant two weeks later. Although this may seem obvious, in the SCI-MT Trial there were instances where a full International Standards for the Neurological Classification of SCI (ISNCSCI) assessment was completed, the participant became unwell, and the site requested randomisation two weeks after the assessment. Using an assessment conducted this far in advance should be avoided, and protocols should clearly specify the maximum acceptable time interval between baseline assessment and randomisation.

#### Schedule outcome assessments relative to randomisation, not the start of the intervention

Scheduling outcome assessments relative to randomisation, rather than the start of the intervention, is essential for ensuring comparability across participants. In the SCI-MT Trial, participants were assessed at 10 weeks and 6 months post-randomisation. Occasionally, the start of the intervention was delayed due to illness or other factors, which showed how assessment timing could become inconsistent if it were tied to the start of the intervention rather than to randomisation. This approach was applied to both the 10-week and 6-month assessments. The protocol also defined acceptable windows for assessments, with assessments conducted within these windows retained for analysis. Assessments falling beyond the maximum allowable windows were not included in the analysis and were therefore counted as missing. Because overall missingness remained below the protocol-specified 5% threshold, no imputation was required. Establishing these rules in advance avoids potential bias.

#### Blind assessors and verify the success of blinding

Blinding assessors is a fundamental requirement in modern clinical trials. This was particularly important for the SCI-MT Trial, where assessors were mainly physiotherapists who may have held pre-existing views about the intervention’s effectiveness. We confirmed these beliefs through a survey circulated to all trial assessors (and other trial personnel), asking them to predict the trial results. Of the 18 assessors who responded, 14 (78%) predicted that the additional Motor Training would increase Total Motor Scores.

We also tested the success of blinding by asking assessors, at the end of each assessment, whether they had been unblinded and, if not, to guess the participant’s group allocation. Including such checks is an important addition to any trial to assess the success of blinding. Assessors can become unblinded in various ways. For example, through an inadvertent comment by a participant or another therapist, or through observing something that reveals group allocation. For example, in our trial, an assessor might have guessed that a participant was in the intervention group if they saw a Practice Sheet near a participant’s bed and knew these were used only by intervention participants. Where feasible, sites should identify a second blinded assessor who can step in if unblinding occurs. To minimise the risk of unblinding, assessors should receive only essential information about the trial, although in practice this can be difficult to achieve.

#### Conceal allocation for those enrolling participants

Concealed allocation is a form of blinding that applies to participant enrolment rather than outcome data collection. It relates to ensuring that individuals making decisions at the site about a participant’s eligibility do not know, and cannot predict, the person’s group assignment [29]. This is typically achieved by having an independent statistician prepare the randomisation schedule and, in our case, upload it to REDCap. If the randomisation schedule uses permuted blocks, it is essential that these blocks vary in size to prevent sites from predicting the assignment of the final participants in each block.

Concealed allocation is critical to ensuring that decisions about inclusion are not influenced by knowledge of group allocation. In the SCI-MT Trial, this was particularly important, as sites might have been tempted to allocate participants perceived as less tolerant of intensive exercise to the control group, and those likely to be disappointed if they missed additional Motor Training to the intervention group. Such allocation guidance could introduce selection bias, as factors other than randomisation dictate group assignment, potentially influencing outcomes [30].

### The intervention

#### Describe the intervention in accordance with the TIDIER Guidelines

For complex interventions, such as in the SCI-MT Trial, it was important that the intervention was described in sufficient detail for others to understand and replicate. There are comprehensive TIDIER Guidelines to guide the reporting of interventions [31]. Sometimes the intervention can be sufficiently explained in the definitive paper with the results or an earlier paper committed to the protocol. However, in our case the intervention was described in a dedicated publication (with a 80-page Supplementary File) [4], to avoid the common criticism that therapy is a Black Box [32].

#### Provide comprehensive training to intervention staff and ensure understanding of key principles

While the SCI-MT Trial was pragmatic and designed to reflect usual clinical practice, intervention staff still required structured training. It was essential that they understood the key principles of the intervention. Therapists were encouraged to apply their clinical reasoning and personalise each intervention for the participant, but it was crucial to ensure that the additional Motor Training intervention was delivered as intended [7]. The training of each therapist was documented and verified during site monitoring visits. No therapist was permitted to administer the intervention until they had completed training. In addition, all sites were provided with recordings of the training for later reference, along with a comprehensive, detailed intervention manual.

#### Monitor intervention delivery to confirm fidelity

It is important to plan in advance how fidelity to the intervention will be assessed in a complex trial. We monitored this in several ways, including auditing trial charts to ensure participants received the 12 h per week of additional Motor Training. We also audited participants’ Practice Sheets, which detailed every exercise they performed. Additionally, we conducted spot audits, where a member of the research team observed therapists administering the intervention and used a checklist to ensure adherence to key principles [7]. In retrospect, auditing sessions at multiple stages would have provided a more complete picture of fidelity, as therapists’ performance likely improved over time. More frequent and earlier spot audits would have provided a fuller picture of fidelity. However, spot audits are difficult and resource-intensive to implement. While it is helpful to understand how the intervention was operationalised throughout the trial, any deviations from the intended protocol likely reflect the deviations that would occur in real-world practice. Because this was a pragmatic trial analysed using intention-to-treat principles, no deviation in intervention delivery was considered grounds for excluding participants or modifying their data. Fidelity monitoring was undertaken to document how the intervention was delivered in practice, rather than to enforce strict adherence. When deviations were identified, they were recorded and reported to provide an accurate description of real-world implementation, as detailed in our published fidelity report [7].

### Data management and trial integrity

#### Confirm randomisation details to prevent allocation errors

Sites may inadvertently treat a participant as belonging to the opposite group to that to which they were randomised, highlighting the need for clear procedures and safeguards. This is most likely to occur soon after the site is informed of the allocation. To prevent such errors, sites should provide written confirmation of the participant’s allocation and retain a copy of the confirmation email in their site file. The site monitor should then verify that there has been no mix-up and that these procedures have been followed. At the end of the trial, the randomisation schedule should be cross-checked against each site’s allocation records.

#### Design the Case Report Forms (CRFs) and database to minimise errors and simplify data entry

The CRF should be designed so that an assessor can move through it seamlessly and logically. Assessors should not need to move back and forth between pages, as this is a common source of errors. Similarly, assessors should never be asked to perform calculations to derive a score; all answers for every assessment and question should be included directly on the CRF. For questionnaires or assessments that involve a list of possible answers, always ensure they are formatted consistently, with higher scores consistently assigned to either the first or last option. Flipping the arrangement of positive and negative answer options between outcomes is a common source of error. If an assessment requires reading recordings of reports, these should be retained as source data. It is equally important that the database mirrors the CRFs, so any information collected on the CRF should also appear in the database. For example, record the date of birth rather than requiring assessors or data-entry staff to calculate and enter age. The same principle applies to any data point used to derive another value. In some cases, data may be entered directly online during the assessment instead of on paper. While this eliminates separate data entry, it can make potential errors more difficult to identify and correct. Although these principles may appear straightforward, they are a common source of preventable error in practice and are often overlooked by trialists who do not routinely design CRFs and databases.

In addition, from the outset, it should be clear how every piece of information collected on a CRF will ultimately be used. This helps prevent unnecessary data collection, and saves time and resources for everyone involved. The most effective approach is to design the Table Shells for the final publication in advance, typically included in the Statistical Analysis Plan (see our SCI-MT SAP for an example [2]). Researchers should then ensure that each item on the CRF can be directly mapped to a corresponding element in the Table Shells [12].

#### Continuously verify data accuracy throughout the trial, not just at the end

All data, including screening logs, CRFs, and, in the case of the SCI-MT Trial, records of every therapy session, must be collected and checked by research staff on a regular basis. This includes checking that responses to questions are consistent with what would be expected from neurological details or answers to other questions. For example, in the SCI-MT Trial responses were occasionally received that were inconsistent with the level of independence expected from the ISNCSCI results. These were queried to check that the assessor had not made a mistake. It is particularly important for a central research staff member to verify eligibility of participants and baseline assessments prior to randomisation so any potential problems that might exclude a person are identified before they are randomised. It is not sufficient to perform only out-of-range or similar checks at the end of the trial. Identifying potential errors at that stage is too late to resolve them. If errors are picked up early, the sites have a chance to resolve data queries. In addition, meticulous records must be kept of every data point queried and any subsequent changes made in response [12]. For example, if a CRF is queried because the date at the top of the form appears incorrect, a data query form must be sent to the site, and the site must respond formally. If an error is corrected, the change must be documented on the original CRF and in the study database. This diligence ensures that every change is fully traceable.

Data captured in written format on CRFs (as opposed to data recorded electronically in real time) should always be double-entered to minimise errors. The more data collected and the more tedious the entry process, the higher the likelihood of mistakes. This risk increases if the person entering the data does not fully understand what they are transcribing. Most data-capture platforms include functionality for double data entry, and this should be incorporated into the trial setup wherever possible. However, some platform features (such as repeating instruments or forms in REDCap [33]) may limit the ability to use double data entry as intended, so these constraints should be considered during database design.

#### Develop clear procedures for recording Serious Adverse Events (SAEs) and Adverse Events (AEs)

Most trialists are aware of the need to record and report SAEs and AEs [34]; however, practical implementation is often more complex than it initially appears. For example, it is important that both groups of participants have the same opportunities for reporting SAEs and AEs. This is essential because the final analysis of SAEs and AEs involves comparing the two groups. However, participants in the intervention group often spend more time with trial or site staff, making their SAEs and AEs more likely to be noticed and recorded. In addition, the protocol must clearly specify whether SAEs and AEs are recorded for the entire duration of the participant’s involvement in the trial or only during the period when they are receiving the intervention. In a drug trial, it would clearly be important to record SAEs and AEs beyond the last dose of the medication. However, in a trial like SCI-MT Trial, we deemed it reasonable to collect SAEs and AEs only during the first 10 weeks of the trial when the additional Motor Training was being delivered. We also sought to avoid overburdening sites by requiring documentation of every AE, as technically every minor medical issue could be considered an AE. This would include events such as blocked catheters, headaches, or pressure injuries - all very common occurrences after SCI. To address this, we pre-specified which types of AEs would be included and which would not using a risk-based approach [34, 35]. We based our approach on our experiences with an earlier trial (Hands On) [36].

#### Maintain strict version control of study documents

Managing a clinical trial requires meticulous attention to detail, particularly with respect to version control of all study-related documents. Although it is ideal to get the initial version of each document to be correct, issues inevitably arise that require updates or corrections. It is essential that every change is carefully managed and documented. Each document should include a version history section at the end, detailing every update and correction. When a new version is issued, sites should mark every page of the previous version as “superseded” and file it in a separate section of their site folders. Particular care must be taken with consent forms: if a new version is required, it is imperative to consult the Ethics Committee to determine whether participants who have already provided consent need to be reconsented. In some cases, this may be necessary even for participants who have already completed the trial. Although version control may seem straightforward, managing multiple evolving documents across sites, ethics committees, and teams is a frequent operational challenge in clinical trials, and small lapses can have significant downstream consequences. We therefore included this principle to highlight a common operational challenge that trialists frequently underestimate.

#### Use a data safety monitoring committee

For large trials, a Data Safety Monitoring Board (DSMB) is generally advisable, with the need determined by trial risk, intervention characteristics, and oversight requirements. The DSMB meets periodically to review the conduct of the trial, ensure adherence to the protocol and monitor AEs and SAEs. Its primarily role is to ensure the safety and welfare of trial participants [37–39]. A DSMB should include members with a complementary mix of expertise. Our DSMB consisted of a statistician, an experienced clinical trialist, a medical clinician-researcher with a background in SCI, a research physiotherapist familiar with the intervention and an epidemiologist. The procedures and responsibilities of the DSMB must be clearly defined in a DSMB Charter. In particular, the Charter must specify whether the DSMB will have access to unblinded data and under what circumstances it may assess potential futility. Any planned between-group comparisons before trial completion must be pre-specified, and the final analysis must adjust for all interim analyses. The results of any interim analyses must remain strictly confidential to DSMB members only.

### The analysis, interpretation and reporting of results

#### Do not perform statistical tests on baseline data

Trialists should include a table of baseline characteristics for each group in the final published paper containing the results. This is provided to describe the groups and allow readers to assess comparability. In a well-randomised trial, it is extremely unlikely that the groups will be meaningfully different at baseline. Significant differences would suggest a potential problem with randomisation. Regardless, it is never appropriate to perform statistical tests solely to demonstrate that the groups are comparable. Altman stated back in 1985: “*Performing a significance test to compare baseline variables is to assess the probability of something having occurred by chance when we know that it did occur by chance. Such a procedure is clearly absurd*”(Pg 126) [40]. These sentiments have been echoed repeatedly ever since by clinical trial experts, yet this practice persists among some researchers. We include it here because, despite being well established, it remains one of the most commonly misapplied principles in published trials and continues to affect the interpretation of baseline tables.

#### It is occasionally acceptable to exclude randomised participants from analysis

In large trials, there may be occasions when participants are initially deemed eligible and randomised, only to later be found ineligible [41]. This can occur due to errors or the emergence of previously unavailable information. For example, in the SCI-MT Trial, one participant was initially classified as having an AIS C lesion and was therefore randomised. It later became apparent when all ISNCSCI sheets were double checked that the participant actually had an AIS B lesion, which would normally be an exclusion criterion. This incorrect classification resulted from a highly technical and somewhat disputed application of the ISNCSCI. Excluding this participant would have been reasonable, but blinded investigators determined that the difference between AIS B and AIS C in this case was subtle and unlikely to affect the outcome. The participant was therefore retained in the trial. Importantly, this decision was made before the participant’s group allocation was known and prior to any analysis of the trial results, and it was declared in the published paper of the results.

#### Pre-specify all subgroup analyses

The first question often asked when a trial such as the SCI-MT Trial fails to demonstrate treatment effectiveness is whether certain subgroups of participants may have responded differently. This issue is closely related to the trial’s inclusion criteria. In other words, when a treatment believed to be effective fails to show benefit, there is a tendency to argue that it may have helped some participants, but the effect was diluted by the inclusion of all. This is often expressed as criticism of the inclusion criteria, highlighting concerns that too broad or too narrow eligibility may have masked or exaggerated treatment effects and contributed to heterogeneity in the trial population. This issue is frequently linked to discussions about responders and non-responders, although they reflect the same underlying concept. The only correct approach to this issue is to anticipate this possibility before starting the trial and pre-specify any subgroup analyses in the trial protocol. There should typically be no more than two or three, and these analyses will inevitably provide imprecise estimates of treatment effects, as the sample size will be smaller for each subgroup than the whole group upon which power analyses were based. It is acceptable to perform post-hoc exploratory subgroup analyses, but these are exactly that: exploratory. It is critical not to overinterpret any subgroup analysis finding, whether pre-specified or post-hoc. Such findings are frequently spurious (i.e., false positives) and can be misleading [25, 42].

#### Interpret mean between-group differences relative to a prespecified minimal important difference

Many researchers fail to report the most important trial outcome: the mean between-group difference for continuous outcomes. This should always be presented in the raw units of measurement rather than as standardised or transformed score. To aid interpretation, the range and direction of the outcome scale should be clearly specified, ideally in the results table, including whether higher or lower values indicate a better outcome. The results for the primary outcome should then be interpreted in relation to a prespecified smallest worthwhile effect [43]. Importantly, the smallest worthwhile effect (also termed the *minimally important treatment effect*) is distinct from the smallest detectable difference [44]. There is no universal smallest worthwhile effect for any outcome; it is specific to the trial context and the intervention under investigation. It is not a statistical concept, so Cohen’s effect sizes are not appropriate for interpreting the magnitude of a treatment effect.

The most robust way to determine the smallest worthwhile effect is through the benefit–harm trade-off method [45]. If this is not feasible, a panel of researchers, clinicians, and people with SCI can agree on a reasonable value after considering the time, cost, and potential harms of the intervention. This value should reflect the *additional* benefit of the intervention beyond improvements expected from natural recovery or usual care. Although the concept can be challenging to communicate, it is fundamental to interpreting trial results. In the SCI-MT Trial, we pre-specified the smallest worthwhile effect initially through consultation with researchers, clinicians and people with SCI. We subsequently verified this value using the benefit–harm trade-off method [6]. Our prespecified value of 6 points on the 100-point total motor score lay midway between the values identified by people with SCI (3 points) and therapists (9 points) which provided additional credibility to our prespecified smallest worthwhile effect. Importantly, the people with SCI recruited to the smallest worthwhile effect study had previously experienced the intensive Motor Training, and the therapists had delivered the intervention. This gave both groups first hand insight into the demands of the intervention and the functional implications of changes in strength.

#### Changes over time within groups do not provide inherently useful information

Any within-group changes over time should not be used to determine treatment effectiveness, as they cannot distinguish intervention effects from natural recovery or other influences. They may, however, be reported to show whether the between-group difference arose from improvement in one group, deterioration in the other, or a combination of both. Statistical improvements in the intervention group alone without a corresponding between-group difference must never be interpreted as evidence of a treatment effect [46]. Likewise, improvements observed in both groups do not provide evidence about the effectiveness of a co-intervention delivered to both groups. For example, readers of the SCI-MT Trial may be tempted to interpret improvements in both groups as indicating that usual care (received by both groups) was effective. This interpretation is not justified. The only way to determine whether usual care is truly effective would be to compare it with no intervention, a study design that would be unethical to conduct.

#### Interim trial results should not be reported except under predefined conditions

It is rarely appropriate to publicly present or report interim results (trial results specifically refer to between-group differences, not to baseline descriptive data or data not reported by group) [47]. Reporting such data indicates that the trialists have examined their results before trial completion. Unless, such analyses have been clearly pre-specified, reporting them is generally considered poor practice. The data analysis should only be performed after the trial is complete and the database has been cleaned and locked. The analysis should then be conducted by blinded statisticians according to a pre-specified and publicly available statistical analysis plan. Any premature examination of results without clear justification and pre-specification indicates that good clinical trial practices have not been followed and raises questions about the integrity of both the data and the analysis.

#### Report all between-group results in a single publication and avoid spin

Clinical trials are labour- and time-intensive and do not always yield large numbers of publications. In today’s academic environment, where publication quantity is often prioritised over quality, researchers may be tempted to divide their trial results into multiple smaller publications to increase output. However, this practice is misleading and unhelpful, as it fragments the interpretation of trial findings [48]. If a justifiable argument can be made for publishing secondary outcomes separately, then each must be clearly interpreted in the context of the primary outcome, and all prior related publications must be explicitly declared. Otherwise, secondary outcomes risk being misinterpreted, as they will not be viewed in light of the primary findings. Failure to follow this principle can also result in the same clinical trial being counted multiple times in meta-analyses, distorting the evidence base.

#### Share trial results with all participants and stakeholders upon trial completion

Finally, ensure that reasonable efforts are made to inform participants about the trial results once the trial is completed. This is a small but important gesture of respect for their time and effort [49].

## Operational principles

### Operational issues

#### Assemble a skilled, collaborative team you trust

The research team is central to the success of a trial, so members should be selected carefully. Each person should bring the necessary skills and expertise, ensuring a balanced mix of academics and clinicians, as well as early-career and experienced researchers. Your team should include a statistician, a health economist, an academic with expertise in process evaluations and qualitative data, and researchers with content expertise [12]. It is equally important to include consumers who can represent the perspectives of people with lived experience of SCI.

Whenever possible, assemble a group of people who have worked together before and who demonstrate a collaborative and constructive approach: people willing to do the hard work to ensure the trial succeeds. Many trials have faltered due to interpersonal conflicts between investigators, so take steps from the outset to prevent this. This includes establishing a clear policy on authorship and roles. As part of the SCI-MT Trial, we asked sites to provide a written and agreed upon policy for authorship from their site.

#### Select sites with similar operations, values and funding models

Just as it is important to take care when selecting your research team, it is also important to give careful consideration to the appropriate sites to include in a trial. For the SCI-MT Trial, it was particularly important to ensure that potential sites kept patients in hospital for at least 20 weeks. This was to ensure that the intervention could be administered for 10 weeks when some participants may not have been randomised until 10 weeks after injury. It was also important to ensure that the included sites provided similar usual care. Of course, usual care will invariably differ between sites and this can be somewhat dealt with by stratifying the randomisation by site (and including site as a covariate in the analysis). Nonetheless, we wanted sites that provided somewhat similar usual care. It was also important to ensure that the therapists delivering usual care were supportive of the trial because they were the ones who were going to be most relied upon to make the trial happen. The trial could not succeed if enthusiasm was limited to the medical specialists.

Finally, it was essential that all sites were funded in a similar way for their usual operations. For example, all the sites in the SCI-MT Trial were funded by the government. Differences in funding models may create variations in operational priorities, which could influence how trial procedures, including randomisation, are implemented. For example, privately funded units may need to carefully account for therapist’s time, making it more difficult for them to provide in-kind support or flexibility. Selecting sites with similar funding models helped ensure consistent implementation of all trial procedures.

#### Be mindful of the additional workload placed on the SCI units

Unless you have the funding to pay for every additional task undertaken by sites as part of your trial, you will inevitably rely to some extent on goodwill. In the SCI-MT Trial, sites were paid to cover the costs of the additional Motor Training, and they received extra payments to cover some additional expenses. However, these payments did not cover everything. For example, usual care therapists were asked to record the details of every physiotherapy and occupational therapy session provided to all trial participants. They were also asked to attend training sessions. These were extra tasks on top of their already busy caseloads. They did not receive formal recognition for this work, nor were they financially compensated beyond their usual salaries. It is therefore important not to build too much additional work into your trial [12]. It is also essential to acknowledge their contributions and foster a workplace culture that values evidence and engagement in research [12].

#### Engage a reputable Clinical Research Organisation (CRO) but read the fine print and expect to pay for the service

Large clinical trials require specialised operational expertise, and research teams may not always have the capacity or experience to manage every component internally. In such cases, engaging a Clinical Research Organisation (CRO) can provide valuable support, particularly for activities such as data management, monitoring, and ensuring adherence to International Council for Harmonisation – Good Clinical Practice (ICH-GCP). However, CROs are commercial enterprises, and professional trial management comes at a substantial cost. It is essential that contracts clearly define responsibilities, deliverables, and expectations, as assumptions about what is included can lead to gaps in service or unanticipated work for the research team. CRO staff may also be more familiar with commercially sponsored trials than investigator-initiated studies, so clear communication and well-defined processes are important to ensure smooth collaboration. Overall, CROs can provide high-quality support, but teams should carefully consider their needs, budget, and internal expertise before deciding whether to engage one.

#### Allow substantial time for contracts and approvals especially with international sites

Obtaining ethical approval for research is often a lengthy process. This process is even slower when navigating international ethics committees and attaining ethical and governance approval for many sites across many countries. Based on our experience with the SCI-MT Trial, these steps required close to 12 months, so trialists should be aware that ethics, governance, and contractual processes may take substantial time and factor this into trial planning. The timeframe will vary across jurisdictions, institutions, and trial types, but our experience illustrates the importance of allowing generous time for approvals and contracting. This is particularly challenging because most granting bodies expect trials to be completed within five years. This is difficult for trials involving people with recent SCI, as recruitment tends to be slow. Including a 12-month follow-up assessment can extend the total duration of the trial to nearly seven years.

#### Understand the country-specific data, consent, and confidentiality regulations

International trials require careful navigation of country-specific rules on data transfer, participant confidentiality, and consent procedures. For example, in the SCI-MT Trial, some countries did not allow collection of participants’ dates of birth. Although this was not a major issue, it added complexity to data collection and analysis. There are also specific consent rules for participants aged between 16 and 18 years, which vary between countries. For instance, some countries require a guardian to sign consent for participants between 16 and 18 years, while others do not. Similarly, there were different rules about what needed to be recorded in participants’ medical notes about their participation in the trial. Furthermore, any data transferred out of a country must be fully de-identified and approved. This can create challenges if a central site in one country is responsible for contacting participants in another country for follow-up assessments. Many similar issues must also be addressed on a case-by-case basis.

#### Be prepared for additional challenges when conducting trials in many languages

The SCI-MT Trial was conducted in four different languages, which introduced a range of challenges. Most staff and investigators at the sites had a good level of English, which helped minimise misunderstandings. However, all patient-facing materials needed to be translated into all four languages, adding considerable cost and complexity. The accuracy of the translation was particularly important for all outcome measures.

In addition, data entry staff did not speak all languages. It was therefore essential that the CRFs and database (in our case, REDCap) displayed both the translated and English versions side by side for every sentence, question, and answer. This facilitated accurate data entry and checking, and provided a straightforward way to identify and correct any translational errors.

## Conclusion

The purpose of this paper is to provide guidance to future trialists on designing and conducting international clinical trials in SCI to the highest possible standards. Drawing on our experiences with the SCI-MT Trial, we have outlined key principles relating to trial design (Methodological Principles) and trial conduct (Operational Principles). Some of our recommendations may be challenging to implement, particularly those with financial implications, whereas others are essential for the rigorous conduct of any clinical trial. Adherence to these principles will help ensure that trials involving people with SCI produce robust, reliable, and trustworthy findings, thereby strengthening the overall quality of clinical research. Advancing trial methodology in this way is a critical step toward improving evidence-based care.

## Data Availability

There are no data associated with this publication.
